# Protocol for measuring cellular energetics through noncanonical amino acid tagging in human peripheral blood and murine tissue immune cells

**DOI:** 10.1016/j.xpro.2025.104134

**Published:** 2025-10-08

**Authors:** Frank Vrieling, Hendrik J.P. van der Zande, Manon Dumont, Rinke Stienstra

**Affiliations:** 1 Division of Human Nutrition and Health, Wageningen University, 6708 WE Wageningen, the Netherlands; 2 Department of Internal Medicine, Radboud University Medical Center, 6525 GA Nijmegen, the Netherlands

**Keywords:** Cell Biology, Cell culture, Cell isolation, Flow Cytometry, Immunology, Metabolism, Molecular Biology, Molecular/Chemical Probes

## Abstract

Cellular metabolism dictates immune cell function, yet we lack tools to functionally profile immunometabolism in low-yield, complex samples. We present a flow cytometry-based protocol for measuring cellular energetics through noncanonical amino acid tagging (CENCAT) in human peripheral blood and murine tissue immune cells. We describe steps for sample preparation, metabolic inhibition, protein synthesis analysis using click chemistry, immunophenotyping, and calculation of metabolic dependencies.

For complete details on the use and execution of this protocol, please refer to Vrieling et al.^1^

## Highlights

• Instructions for preparing human PBMCs and murine tissues for metabolic profiling
• Protocol for βES incorporation in nascent proteins under metabolic inhibition
• Step-by-step guide to fluorescent tagging of nascent proteins via click chemistry
• Procedure for calculating relative cellular glucose and mitochondrial dependence

## Abstract (editor highlights)

Publisher’s note: Undertaking any experimental protocol requires adherence to local institutional guidelines for laboratory safety and ethics.

## Graphical abstract

**Figure:**
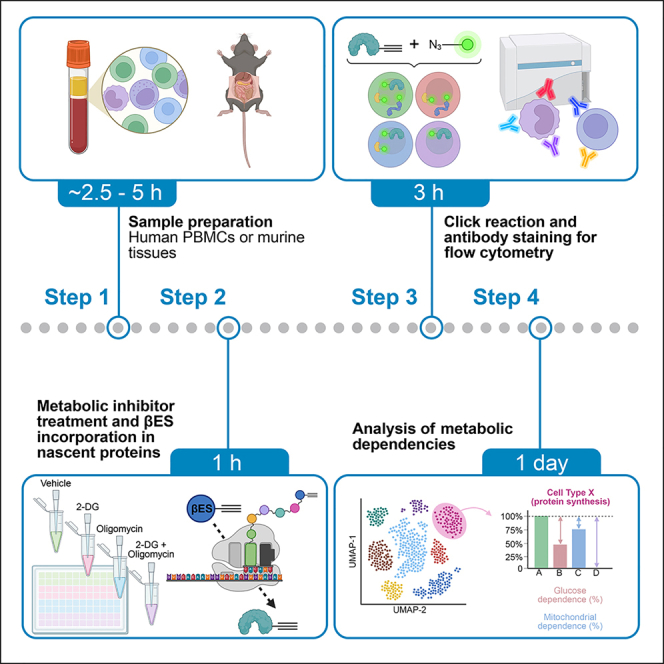

## Before you begin

This protocol describes the profiling of cellular metabolism using the flow cytometry-based tool CENCAT. We have used this protocol for determining metabolic profiles of naïve and activated human peripheral blood mononuclear cells (PBMCs) and murine tissue-resident immune cells from the peritoneal cavity, spleen, epidydimal white adipose tissue, liver, lungs and kidneys.^1^ This ‘before you begin’ section contains multiple brief workflows for isolating these cells. The isolation procedures are not unique to this protocol and are based on previous work.^2^^,^^3^ However, alternative approaches can also be applied (e.g.^4^^,^^5^^,^^6^). Since CENCAT allows the use of any live single cell suspension that can be acquired using flow cytometry, cells from other species, tissues or (non-immune) lineages of interest may be used instead. Finally, given that CENCAT is a flow cytometry-based tool, both homogeneous and heterogeneous suspensions can be metabolically profiled.

Experience with multicolor flow cytometry is necessary, yet this falls beyond the scope of this protocol. Design and execution of high parameter flow cytometry have been discussed elsewhere.^7^ We provide antibody panels for detecting most major immune lineages in human PBMCs and mouse tissues, yet the user may change the panel as desired. However, antibody titration, testing for epitope recognition after formaldehyde fixation and resistance to fluorophore quenching by copper-catalyzed azide–alkyne cycloaddition (CuAAC) should be considered when designing an adjusted panel. Of note, different antibody batches and flow cytometer hardware may impact staining resolution. Thus, we recommend the antibody panels shown in this protocol are tested and optimized by the user.

### Innovation

Extracellular flux (XF) analysis has long been the cornerstone of immunometabolism research. In XF analysis, the relative rates of cellular oxygen consumption rate (OCR) and extracellular acidification rate (ECAR) can be used to infer glycolytic and oxidative metabolism. Limitations of XF analysis include the need for cell purification, relatively large cell numbers and specialized equipment. To overcome this, a method called SCENITH (single-cell energetic metabolism by profiling translation inhibition) was developed, a flow cytometry-based assay which quantifies protein synthesis inhibition as a proxy for metabolic activity. While powerful, SCENITH relies on intracellular detection of puromycin, a toxic antibiotic that can perturb cellular physiology during the assay.

The method presented here, CENCAT, is an innovative approach that integrates biorthogonal noncanonical amino acid tagging (BONCAT) into a SCENITH-like workflow for assessing metabolic activity. BONCAT enables the incorporation and detection of non-toxic noncanonical amino acid incorporation into nascent proteins, thereby avoiding the use of puromycin. This strategy preserves cell physiology during the assay while maintaining the advantages of the original protocol compared to XF analysis. By combining the accessibility of flow cytometry with the specificity of BONCAT, our method provides a less disruptive, versatile alternative to existing approaches for profiling immune cell metabolism.

### Institutional permissions

All blood samples were collected after acquiring written informed consent as per the norms of the International Declaration of Helsinki. Ethical approval was obtained from the Medical Ethical Committee Oost Nederland (NL84281.091.23). Animal experiments followed the Guide for the Care and Use of Laboratory Animals of the Institute for Laboratory Animal Research and were approved by the Central Authority for Scientific Procedures on Animals (CCD, AVD10400202115283) and the Institutional Animal Care and Use Committee of Wageningen University.

Users of this protocol must acquire relevant ethical approval for conducting their experiments.

### Preparation of required solutions

**Timing: variable**

***Note:*** Recipes for required solutions are detailed under materials and equipment.

1. Prepare solutions, buffers and stocks required for immune cell isolation:
  a. Peritoneal lavage buffer.
  b. Krebs buffer.
  c. HEPES stock.
  d. HEPES-buffered Krebs solution.
  e. Collagenase D stock.
  f. DNAse I stock.
  g. eWAT digestion buffer.
  h. Liver digestion buffer.
  i. Spleen digestion buffer.
  j. Kidney/lung digestion buffer.
  k. Wash buffer.
  l. RBC lysis buffer.
2. Prepare solutions, buffers and stocks required for CENCAT:
  a. Complete RPMI1640 medium.
  b. 2-DG stock.
  c. Oligomycin stock.
  d. βES stock.
  e. Cu(II)SO_4_ stock.
  f. THPTA stock.
  g. AZDye Azide Plus stock.
  h. Permeabilization buffer.
  i. Click buffer.
  j. FACS buffer.

### Human PBMC isolation

**Timing: 1.5 h**

The following steps describe the isolation and subsequent activation of human peripheral blood mononuclear cells (PBMCs) from EDTA blood using Ficoll density centrifugation. Other methods to isolate the PBMC fraction from peripheral blood can be applied as well.

3. Dilute EDTA blood 1:1 with PBS at 18°C–20°C.
4. Divide and carefully layer the diluted blood on the porous barrier of the Leucosep tubes containing Ficoll Paque-Plus.

**CRITICAL:** Do not pipette directly on top of the porous barrier but hold the tube at a 45-degree angle and pipette the blood against the side.

5. Centrifuge Leucosep tubes at 800 RCF for 15 min at 18°C–20°C.
6. Transfer the top layer containing the PBMC fraction of each Leucosep tube to a fresh 50 ml tube by pouring or pipetting.
7. Add ice-cold PBS each tube until 50 ml mark and invert tubes three times.
8. Centrifuge tubes at 300 RCF for 10 min at 4°C.
9. Carefully discard supernatant using aspiration and resuspend the cell pellet.

***Optional:*** Users may choose to pool the PBMCs of multiple tubes into one at this point in time.

10. Repeat washing steps 7–9 two more times.
11. Count the PBMCs using a hemocytometer or any type of automated cell counter.

### PBMC activation or resting

**Timing: 2 h**

To illustrate the utility of CENCAT, PBMCs were activated using lipopolysaccharide (LPS) or TransAct (synthetic CD3/CD28 agonist) for 2 h or left unstimulated.

12. Dilute PBMCs to 10.0 × 10^6^ cells/mL in complete RPMI1640 medium.

***Note:*** The working range of PBMCs for CENCAT is 0.25–1.0 × 10^6^ cells/well, which equals a total cell requirement of 1.0–4.0 × 10^6^ per replicate/condition as a minimum of four wells is required for a single measurement (Vehicle, 2-DG, oligomycin, 2-DG + oligomycin). We recommend the inclusion of technical duplicates for each treatment condition if possible.

13. Add 1.0–4.0×10^6^ PBMCs to sterile polypropylene FACS tubes (one per condition).
14. Add the required stimulations:
  a. Unstimulated.
  b. LPS: 10 ng/mL.
  c. TransAct: 1:100 dilution.
15. Gently mix samples by pipetting and incubate tubes for 2 h at 37°C/5%CO_2_.

**CRITICAL:** If no activation steps are applied, rest the PBMCs for 1 h at 37°C/5%CO_2_ before initiating CENCAT to allow cells time to recover metabolically from the isolation procedure.

16. Resuspend the cells in the FACS tubes by pipetting.
17. Plate 0.25–1.0 × 10^6^ cells per replicate in 90 μL in a 96-well round-bottom plate. Four wells are required for each replicate, one for each inhibitor condition.
18. Continue with CENCAT.

### Murine tissue sampling and processing

**Timing: 5 h**

The following steps describe the sampling and processing of tissues from mice for immune cell isolation and CENCAT. Timing is indicative for processing of tissues from three mice by one researcher. Since the protocol includes incubation steps, the tissues can be processed in parallel, reducing the time required.

19. Euthanize mice by cervical dislocation.

***Optional:*** Users may choose to terminally anesthetize mice and collect blood for immune cell isolation prior to cervical dislocation.

20. Collect the peritoneal lavage.
  a. Immediately after cervical dislocation, open the skin by making a small incision and rupture the skin to expose the abdominal wall and inject 10 mL peritoneal lavage buffer using a 10-mL syringe.
  b. Remove the needle and gently shake the mouse for 30 s to release peritoneal immune cells.
  c. Retrieve as much volume as possible from the peritoneum using the same syringe, collect in a 15-mL tube and keep on ice.

**CRITICAL:** Make sure to leave peritoneum intact before injecting peritoneal lavage buffer, otherwise peritoneal cells may leak out. In addition, take care not to puncture blood vessels or the intestine with the needle when collecting the peritoneal wash, to prevent contamination with circulating immune cells or the microbiome, as also detailed elsewhere.^8^

21. Cut the peritoneum and open the thorax, exposing abdominal and thoracic organs.
22. Harvest epidydimal white adipose tissue (eWAT) by cutting along the testes and removing the epididymis.
  a. Collect in a 50-mL tube filled with 10 mL RPMI-1640. Keep at 18°C–20°C.
23. Harvest the liver and remove the gall bladder. Harvest the spleen, kidneys and lungs.
  a. Collect in 50-mL tubes filled with 10 mL RPMI-1640. Keep on ice.

***Optional:*** Users may choose to transcardially perfuse mice with 10 mL PBS using a 10-mL syringe to remove circulating immune cells from tissue vasculature.

**Figure 1:**
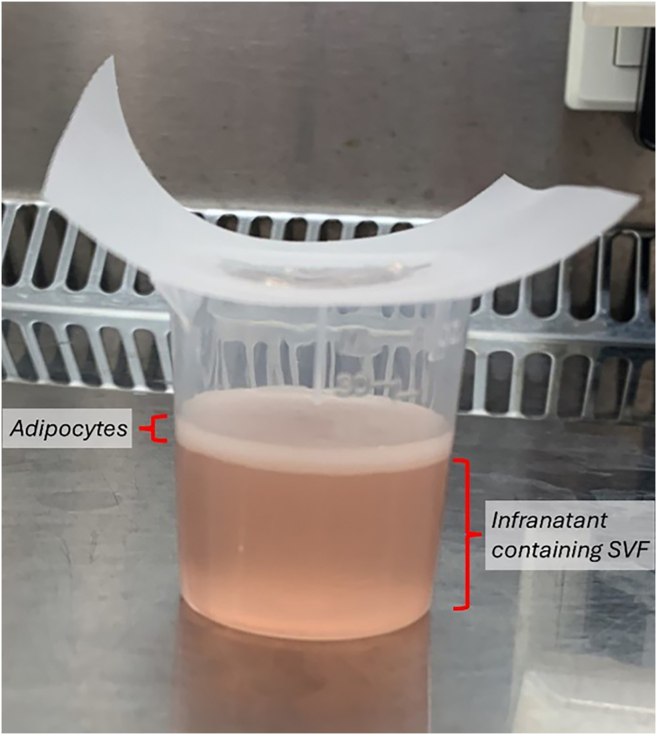
Separation of adipocytes from stromal vascular fraction using 30 mL beakers and 250 μm Nitex filters

24. Peritoneal lavage processing.
  a. Filter peritoneal lavage through a 40 μm cell strainer into a new 50-mL tube.
  b. Wash 15-mL collection tube with 10 mL ice-cold wash buffer and pass through strainer.
  c. Wash strainer with wash buffer up to a total volume of 40 mL.
  d. Centrifuge at 400 RCF for 5 min at 4°C. Discard supernatant.
  e. Count cells using a hemocytometer or any type of automated cell counter.
  f. Plate ≥250,000 cells per replicate in 200 μL complete RPMI1640 medium in a 96-well round-bottom plate. Four wells are required for each replicate, one for each inhibitor condition.
    ***Note:*** As the working range of murine tissue immune cells for CENCAT is 0.25–1.0×10^6^ cells/well, 1.0–4.0×10^6^ cells are required per replicate. See the “cell yield from murine tissues” for expected immune cell yields from murine tissues. In case of lower cell yield per mouse, pool cells of multiple mice in the same experimental group. Lower numbers of cells per replicate are not recommended for heterogeneous populations, as at least 500 cells per subset are required for reliable measurement. We recommend the inclusion of technical duplicates for each treatment condition if cell counts allow.
  g. Rest the cells for 1 h at 37°C/5% CO_2_ before initiating CENCAT.
25. eWAT processing.
  ***Note:*** Buffers for isolation of immune cells from eWAT should be at 18°C–20°C to prevent freezing of lipids.
  a. Transfer fat pads from 50-mL tube to a 100-mm petri dish using a forceps.
  b. Use single-edged razors to mince tissue into small pieces (∼2 mm^2^ pieces).
    i. Use two razors to pull big pieces of tissue apart.
    ii. Add 1 mL Krebs/HEPES/BSA buffer and collect minced tissue in a 50-mL tube containing 1.5 mL Krebs/HEPES/BSA buffer.
  c. Add 2.5 mL 2× concentrated eWAT digestion buffer to 50-mL tube.
  d. Incubate at 37°C/5% CO_2_ for 45 min under agitation on a shaker platform (100 rpm).
    ***Note:*** We sampled tissues from adult (12–16 weeks old) naive C57BL/6J mice. Volume of digestion buffers may require optimization for mice with larger or smaller fat pads.
  e. Inhibit digestion by adding 5 mL wash buffer.
  f. Transfer digested tissue into a 30 mL polypropylene beaker through a 250 μm filter (Figure 1) using a 10-mL serological pipette.
    i. Use the rubber-end of the plunger of a 3-mL syringe to gently push cells through the filter.
    ii. Wash 50-mL digestion tube with 10 mL wash buffer.
    iii. Wash filter twice with 5 mL wash buffer (use plunger to gently push digest through the filter).
      **CRITICAL:** Transfer the digested tissue onto the filter in small portions to avoid filter overflow upon exceeding the filter capacity.
      ***Note:*** A 100 μm cell strainer is not advised for filtering adipose tissue digests, since the diameter of adipocytes may exceed 100 μm, increasing the risk of adipocyte rupture. Release of ruptured adipocytes contents may result in staining artifacts in flow cytometry analysis, especially when performing formaldehyde fixation.
  g. Allow adipocytes to surface by resting the sample for 5 min at 18°C–20°C.
  h. Take the infranatant using a 20-mL syringe with 18-G blunt-end needle (Figure 1), and collect this stromal vascular fraction (SVF) in a 50-mL tube.
    ***Note:*** Continue the isolation at 4°C or on ice from here onwards, when adipocytes are separated from the stromal vascular fraction.
  i. Pellet the SVF at 350 RCF for 5 min at 4°C. Discard supernatant.
  j. Resuspend cells in 1 mL ice-cold RBC lysis buffer.
    i. Incubate 2 min at 18°C–20°C while regularly mixing tube.
    ii. Stop reaction by adding 10 mL ice-cold wash buffer.
  k. Filter through 40 μm cell strainer as for peritoneal lavage.
  l. Pellet the cells and discard supernatant (step i).
  m. Count cells using a hemocytometer or any type of automated cell counter.
  n. Plate ≥250,000 cells per replicate in 200 μL complete RPMI1640 medium in a 96-well round-bottom plate. Four wells are required for each replicate, one for each inhibitor condition. Inclusion of technical duplicates is recommended if cell counts allow.
  o. Rest the cells for 1 h at 37°C/5% CO_2_ before initiating CENCAT.
26. Liver processing.
  ***Note:*** The described protocol is sufficient to isolate all major immune cell subsets from the liver. However, in order to maximize the yield of Kupffer cells, *in vivo* perfusion of the liver using a collagenase/dispase digestion mix is required, as described elsewhere.^9^
  a. Transfer liver from 50-mL tube to a 100-mm petri dish using a forceps.
  b. Use single-edged razors to mince tissue into small pieces (∼2 mm^2^ pieces).
    i. Use two razors to pull big pieces of tissue apart.
    ii. Collect minced tissue in a 50-mL tube containing 5 mL RPMI-1640.
  c. Add 5 mL 2× concentrated Liver digestion buffer to 50-mL tube.
  d. Incubate at 37°C/5%CO_2_ for 25 min under agitation on a shaker platform (100 rpm).
    ***Note:*** We sampled tissues from adult (12–16 weeks old) naive C57BL/6J mice. Volume of digestion buffers may require optimization for mice with steatotic and/or fibrotic livers.
  e. Put tubes on ice.
  f. Mix tubes and pour digest into a new 50-mL tube through a 100 μm cell strainer.
    i. Use the back-end of the plunger of a 1-mL syringe to push digest through strainer.
    ii. Wash the digest tube with 10 mL ice-cold wash buffer and pass through strainer.
    iii. Wash strainer with wash buffer up to a total volume of 40 mL.
      **CRITICAL:** Pour digested tissue in steps to prevent solution from overflowing the strainer. Lift strainer slightly to facilitate fluid passing through the strainer.
  g. Pellet the cells at 300 RCF for 5 min at 4°C. Carefully discard supernatant by pouring into a waste bottle.
    **CRITICAL:** The pellet is loose at this point. Be careful not to discard the pellet, and pour in one fluid movement without disturbing the pellet.
  h. Resuspend the pellet and add ice-cold wash buffer up to a total volume of 40 mL.
  i. Pellet the cells and carefully discard supernatant (step g).
  j. Perform RBC lysis and filtering through 40 μm cell strainer as for eWAT, but using 3 mL ice-cold RBC lysis buffer.
  k. Stop reaction by adding 30 mL ice-cold wash buffer. Pellet the cells and carefully discard supernatant.
  l. Continue with leukocyte enrichment via CD45 magnetic-assisted cell sorting (MACS; step 29).
27. Spleen processing.
  a. Grind spleen in the 24-well plate containing 1 mL RPMI-1640 using the back end of the plunger of a 1-mL syringe.
  b. Add 1 mL 2× concentrated Spleen digestion mix.
  c. Incubate at 37°C/5%CO_2_ for 25 min under agitation on a shaker platform (100 rpm).
  d. Put plate on ice.
  e. Resuspend digest and transfer into a new 50-mL tube through a 100 μm cell strainer using a 1-mL pipette.
    i. Use the back-end of the plunger of a 1-mL syringe to push digest through strainer.
    ii. Wash well with 1 mL wash buffer and pass through strainer.
    iii. Wash strainer with wash buffer up to a total volume of 40 mL.
  f. Centrifuge at 400 RCF for 5 min at 4°C. Discard supernatant.
  g. Perform RBC lysis and filtering through 40 μm cell strainer as for eWAT, but using 10 mL ice-cold RBC lysis buffer.
    i. Stop reaction by adding 40 mL ice-cold wash buffer.
  h. Centrifuge at 400 RCF for 5 min at 4°C. Discard supernatant.
  i. Count cells using a hemocytometer or any type of automated cell counter.
  j. Plate 1,000,000 cells per replicate in 200 μL complete RPMI1640 medium in a 96-well round-bottom plate. Four wells are required for each replicate, one for each inhibitor condition. Inclusion of technical duplicates is recommended if cell counts allow.
  k. Rest the cells for 1 h at 37°C/5% CO_2_ before initiating CENCAT.
28. Kidney and lung processing.
  a. Transfer tissue from 24-well plate or 50-mL tube to a 100-mm petri dish using a forceps.
  b. Use single-edged razors to mince tissue into small pieces (∼2 mm^2^ pieces).
    i. Use two razors to pull big pieces of tissue apart.
    ii. Collect minced tissue in a 50-mL tube containing 2.5 mL RPMI-1640.
  c. Add 2.5 mL 2× concentrated Kidney/Lung digestion buffer to 50-mL tube.
  d. Incubate at 37°C/5%CO_2_ for 30 min under agitation on a shaker platform (100 rpm).
  e. Put tubes on ice.
  f. Mix tubes and pour digest into a new 50-mL tube through a 100 μm cell strainer.
    i. Use the back-end of the plunger of a 1-mL syringe to push digest through strainer.
    ii. Wash the 50-mL digest tube with 10 mL wash buffer and pass through strainer.
    iii. Wash strainer with wash buffer up to a total volume of 40 mL.
  g. Centrifuge at 400 RCF for 5 min at 4°C. Discard supernatant.
  h. Perform RBC lysis and filtering through 40 μm cell strainer as for eWAT, while using 3 mL ice-cold RBC lysis buffer.
    i. Stop reaction by adding 30 mL ice-cold wash buffer.
  i. Centrifuge at 400 RCF for 5 min at 4°C. Discard supernatant.
  j. Continue with CD45 MACS isolation (step 29).
29. CD45+ MACS isolation (liver, kidney and lung).
  ***Note:*** Leukocyte purification is recommended for tissue digests with low leukocyte purity, since βES incorporation is reduced in low-purity samples. The following steps are largely in accordance with the manufacturer’s protocol (Miltenyi CD45 MicroBeads mouse), with minor modifications since reliable determination of cell yield before purification is not possible.
  a. Add 35 μL CD45 microbeads per sample directly to cell pellet and resuspend the pellet using a 1 mL pipet. Incubate for 15 min at 4°C.
  b. Add 10 mL wash buffer and centrifuge at 300 RCF for 5 min at 4°C.
  c. Carefully remove all the supernatant using a 10-mL serological pipette.
  d. Resuspend the pellet in 10 mL wash buffer.
  e. Place large size (LS) column in a MACS separator and wash column with 3 mL wash buffer.
  f. Transfer cells to column.
    ***Note:*** Transfer in steps, as the capacity of LS columns is ∼5 mL. Wait until column reservoir is empty before proceeding to the next step.
  g. Wash 50-mL tube with 3 mL wash buffer and load on column.
  h. Wash column twice with 3 mL wash buffer.
  i. Remove column from separator, place it on top of a 15-mL collection tube, add 5 mL wash buffer and flush out leukocytes by pushing the plunger into the column.
  j. Centrifuge at 400 RCF for 5 min at 4°C. Discard supernatant.
  k. Count cells using a hemocytometer or any type of automated cell counter.
  l. Plate ≥500,000 cells per replicate for liver and lung, and ≥250,000 cells per replicate for kidney in 200 μL complete RPMI1640 medium in a 96-well round-bottom plate. Four wells are required for each replicate, one for each inhibitor condition. Inclusion of technical duplicates is recommended if cell counts allow.
  m. Rest the cells for 1 h at 37°C/5% CO_2_ before initiating CENCAT.

## Key resources table

**Table:** 

REAGENT or RESOURCE	SOURCE	IDENTIFIER
**Antibodies**		

Anti-human CD14-BUV395 (clone MφP-9); working dilution: 1:200	BD Biosciences	Cat#563561; RRID: AB_2744288
Anti-human HLA-DR-BUV661 (clone G46-6); working dilution: 1:200	BD Biosciences	Cat#612980; RRID: AB_2870252
Anti-human CD56-BUV737 (clone NCAM16.2); working dilution: 1:800	BD Biosciences	Cat#612766; RRID: AB_2813880
Anti-human CD19-APC-R700 (clone HIB19); working dilution: 1:400	BD Biosciences	Cat#564977; RRID: AB_2744308
Anti-mouse CLEC2-FITC (clone 17D9); working dilution: 1:200	Bio-Rad	Cat#MCA5700F; RRID: AB_11152598
Anti-mouse/human CD45R/B220-BV510 (clone RA3-6B2); working dilution: 1:100	BioLegend	Cat#103248; RRID: AB_2650679
Anti-mouse CD4-FITC (clone GK1.5); working dilution: 1:200	BioLegend	Cat#100405; RRID: AB_312690
Anti-mouse CD8a-PE-Cy7 (clone 53–6.7); working dilution: 1:400	BioLegend	Cat#100721; RRID: AB_312760
Anti-mouse/human CD11b-BV650 (clone M1/70); working dilution: 1:800	BioLegend	Cat#101259; RRID: AB_2566568
Anti-mouse CD11c-BV605 (clone N418); working dilution: 1:100	BioLegend	Cat#117333; RRID: AB_11204262
Anti-mouse CD45-PerCP-Cy5.5 (clone 30-F11); working dilution: 1:400	BioLegend	Cat#103131; RRID: AB_893344
Anti-mouse CD64-APC (clone X54-5/7.1); working dilution: 1:400	BioLegend	Cat#139305; RRID: AB_11219205
Anti-mouse F4/80-FITC (clone BM8); working dilution: 1:200	BioLegend	Cat#123108; RRID: AB_893502
Anti-mouse Ly-6C-Alexa Fluor 700 (clone HK1.4); working dilution: 1:400	BioLegend	Cat#128024; RRID: AB_10643270
Anti-mouse I-A/I-E (MHCII)-BV785 (clone M5/114.15.2); working dilution: 1:400	BioLegend	Cat#107645; RRID: AB_2565977
Anti-mouse CD170 (Siglec-F)-PE-Dazzle594 (clone S17007L); working dilution: 1:100	BioLegend	Cat#155529; RRID: AB_2890716
Anti-mouse TIM4-PE (clone RMT4-54); working dilution: 1:200 (1:4000 for peritoneal wash)	BioLegend	Cat#130005; RRID: AB_1227807
Anti-human CD4-Spark Violet 500 (clone SK3); working dilution: 1:400	BioLegend	Cat#344690; RRID: AB_2936686
Anti-human CD8-BV650 (clone SK1); working dilution: 1:200	BioLegend	Cat#344730; RRID: AB_2564510
Anti-human CD62L-Alexa Fluor 488 (clone DREG-56); working dilution: 1:200	BioLegend	Cat#304816; RRID: AB_528857
Anti-human CD16-APC-Cy7 (clone 3G8); working dilution: 1:400	BioLegend	Cat#302018; RRID: AB_314218
Anti-human CD45RA-PerCP (clone HI100); working dilution: 1:400	BioLegend	Cat#304156; RRID: AB_2616997
Anti-human CD123-PE (clone 6H6); working dilution: 1:1600	BioLegend	Cat#306006; RRID: AB_314580
Anti-human CD11c-PE-Cy7 (clone S-HCL-3); working dilution: 1:800	BioLegend	Cat#371507; RRID: AB_2650779
Anti-human TCRγ/δ-APC (clone 11F2); working dilution: 1:400	Miltenyi Biotec	Cat#130-113-500; RRID: AB_2733463
Anti-human CD3-NovaFluor Blue 610-70S (clone UCHT1); working dilution: 1:200	Thermo Fisher Scientific	Cat#H002T03B06; RRID: AB_2896419

**Chemicals, peptides, and recombinant proteins**		

Brilliant Stain Buffer Plus	BD Biosciences	Cat#566385
ViaKrome 808 Fixable Viability Dye	Beckman Coulter	Cat#C36628
Human TruStain FcX	BioLegend	Cat#422302
Mouse TruStain FcX	BioLegend	Cat#101320
True-Stain Monocyte Blocker	BioLegend	Cat#426103
Zombie NIR Fixable Viability Kit	BioLegend	Cat#423106
Zombie Aqua Fixable Viability Kit	BioLegend	Cat#423102
Brefeldin A Solution (1,000×)	BioLegend	Cat#420601
CF820 Succinimidyl Ester	Biotium	Cat#96068
Fetal calf serum (FCS)	Biowest	Cat#S1300
β-ethynylserine-HCl (βES)	Bonger Lab, Leiden Academic Center for Drug Research	N/A
Penicillin-Streptomycin Solution	Corning	Cat#30-002-CI
2-deoxy-D-glucose (2-DG)	Merck	Cat#D8375
Collagenase from *Clostridium histolyticum*, type II	Merck	Cat#C6885
Collagenase from *Clostridium histolyticum*, type V	Merck	Cat#C9263
Collagenase from *Clostridium histolyticum*, type D	Merck	Cat#11088866001
Dispase II	Merck	Cat#D4693
DNase I	Merck	Cat#4536282001
Ficoll Paque Plus	Merck	Cat#GE17-1440-03
Lipopolysaccharide from *Escherichia coli* O55:B5	Merck	Cat#L6529
Oligomycin A	Merck	Cat#75351
THPTA	Merck	Cat#762342
T cell TransAct, human	Miltenyi Biotec	Cat#130-128-758
16% Formaldehyde (w/v), methanol-free	Thermo Fisher Scientific	Cat#28908
GlutaMAX	Thermo Fisher Scientific	Cat#35050061
RPMI 1640 medium (with sodium bicarbonate, without L-glutamine and HEPES)	Thermo Fisher Scientific	Cat#21870076
CellBlox Blocking Buffer	Thermo Fisher Scientific	Cat#B001T03F01
AZDye 405 Azide Plus	Vector Laboratories	Cat#CCT-1474
AZDye 488 Azide Plus	Vector Laboratories	Cat#CCT-1475

**Experimental models: Organisms/strains**		

Mouse: C57BL/6J (B6), 12–16 weeks old, male	The Jackson Laboratory	RRID:IMSR_JAX:000664
Human PBMCs from healthy volunteers, *n* = 6, aged 20–40 years, mix female/male	RadboudUMC	N/A

**Software and algorithms**		

FlowJo software version 10.8.1	BD Biosciences	https://www.flowjo.com/
OMIQ	Dotmatics	https://www.omiq.ai
R version 4.2.2	R Core Team	https://www.r-project.org/
GraphPad Prism software version 8.01	Dotmatics	https://www.graphpad.com/features
ggplot2 version 3.4.22	Wickham et al.^10^	https://cran.r-project.org/web/packages/ggplot2/index.html
cowplot version 1.1.1	Wilke et al.^11^	https://cran.r-project.org/web/packages/cowplot/index.html
ggh4x version 0.2.4	van den Brand et al.^12^	https://cran.rstudio.com/web/packages/ggh4x/index.html
mixOmics version 6.23.4	Rohart et al.^13^	https://www.bioconductor.org/packages/release/bioc/html/mixOmics.html

**Other**		

CytoFLEX Flow cytometer	Beckman Coulter	CytoFLEX
CytoFLEX LX Flow cyotmeter	Beckman Coulter	CytoFLEX
Leucosep tubes	Greiner Bio-One	Cat#227288
CD45 MicroBeads, mouse	Miltenyi Biotec	Cat#130-052-301
LS columns	Miltenyi Biotec	Cat#130-042-401
100 μm cell strainers	PluriSelect	Cat#43-57100-51
40 μm cell strainers	PluriSelect	Cat#43-57040-51
250 μm Nitex filter	Sefar	Cat#03-250/50
30 mL beakers	VWR	Cat#213-3916

## Materials and equipment

### Peritoneal lavage buffer

Per mouse, add 40 μL 0.5 M EDTA solution to 9.96 mL PBS to obtain a 2 mM EDTA/PBS solution. Prepare fresh.

### Krebs buffer

**Table:** For a 5× mix

Reagent	Final concentration	Amount
NaCl	0.6 M	3.5 g
KCl	24 mM	0.179 g
KH_2_PO_4_	6 mM	0.082 g
MgSO_4_	6 mM	0.072 g
ddH_2_O	N/A	100 mL
**Total**	**N/A**	**100 mL**

First dissolve all in 80 mL ddH_2_O and adjust pH to 7.4 using 1 M NaOH. Then add ddH_2_O up to 100 mL and pass through 0.22 μm filter. 5× Krebs buffer can be stored at 4°C for several months.

### HEPES stock

For a 1 M HEPES stock, 11.9 g HEPES in 40 mL ddH_2_O. Adjust pH to 7.4 using 1 M NaOH, add up to 50 mL with ddH_2_O and pass through 0.22 μm filter. 1 M HEPES can be stored at 4°C for several months.

### HEPES-buffered Krebs solution

**Table:** Per mouse

Reagent	Final concentration	Amount
5× Krebs buffer	1×	1 mL
1 M HEPES stock	100 mM	0.5 mL
ddH_2_O	N/A	3.5 mL
**Total**	**N/A**	**5 mL**

HEPES-buffered Krebs solution can be stored at 4°C for several months.

### Collagenase D stock

For a 100 mg/mL collagenase D stock, dissolve 500 mg collagenase D in 5 mL RPMI-1640. Store at −20°C in aliquots for up to 6 months.

### DNase I stock

For a 10,000 kunitz U/mL DNAse I stock, dissolve 10,000 kunitz Units DNAse I in 1 mL ddH_2_O, store at −20°C in aliquots and use within 1 month.

### eWAT digestion buffer

Per mouse first dissolve 100 mg BSA (final concentration 40 mg/mL) in 2.5 mL HEPES-buffered Krebs solution and pass through 0.22 μm filter.

**Table:** Then, per mouse for a 2× mix

Reagent	Final concentration	Amount
Collagenase II	2 mg/mL	5 mg
1 M D-Glucose	12 mM	30 μL
HEPES-buffered Krebs solution + BSA	N/A	2.47 mL
**Total**	**N/A**	**2.5 mL**

Always prepare fresh and dilute twice in minced eWAT.

### Liver digestion buffer

**Table:** Per mouse for a 2× mix

Reagent	Final concentration	Amount
Collagenase V	2 mg/mL	10 mg
Dispase II	2 mg/mL	10 mg
Collagenase D stock solution	2 mg/mL	100 μL
DNAse I stock solution	60 kunitz U/mL	30 μL
RPMI1640	N/A	4.87 mL
**Total**	**N/A**	**5 mL**

Always prepare fresh and dilute twice in minced liver.

### Spleen digestion buffer

**Table:** Per mouse for a 2× mix

Reagent	Final concentration	Amount
Collagenase D stock solution	2 mg/mL	20 μL
DNAse I stock solution	60 kunitz U/mL	6 μL
RPMI1640	N/A	974 μL
**Total**	**N/A**	**1000 μL**

Always prepare fresh and dilute twice in ground spleen.

### Kidney/lung digestion buffer

**Table:** Per mouse for a 2× mix

Reagent	Final concentration	Amount
Collagenase V	2 mg/mL	10 mg
Dispase II	2 mg/mL	10 mg
DNAse I stock solution	60 kunitz U/mL	30 μL
RPMI1640	N/A	4.97 mL
**Total**	**N/A**	**5 mL**

Always prepare fresh and dilute twice in minced kidney/lung.

**Table:** Wash buffer

Reagent	Final concentration	Amount
FCS	1%	5 mL
0.5 M EDTA	2.5 mM	2.5 mL
Sterile PBS	N/A	500 mL
**Total**	**N/A**	**507.5 mL**

Prepare sterile in a flow cabinet. Buffer can be stored at 4°C for up to 3 months.

**Table:** RBC lysis buffer

Reagent	Final concentration	Amount
NH_4_Cl	150 mM	8.02 g
KHCO_3_	10 mM	1 g
Na_2_-EDTA	0.1 mM	37.2 mg
ddH_2_O	N/A	1 L
**Total**	**N/A**	**1 L**

First dissolve all in 800 mL ddH_2_O and adjust pH to 7.2 using 1 M NaOH. Then add ddH_2_O up to 1000 mL. Pass through 0.22 μm filter. Buffer can be stored at 4°C for several months.

**Table:** Complete RPMI1640 medium

Reagent	Final concentration	Amount
FCS	8.9%^a^	50 mL
100× GlutaMAX	1×	5 mL
100× penicillin/streptomycin	1×	5 mL
RPMI1640	N/A	500 mL
**Total**	**N/A**	**560 mL**

a Commonly referred to as 10% FCS. Medium can be stored at 4°C for up to 3 months.

### 2-DG stock

For a 2 M 2-deoxy-D-Glucose (2-DG) stock, dissolve 5 g 2-DG in 15.23 mL ddH_2_O. Prepare aliquots and store at −20°C. Stock can be used for up to 6 months.

### Oligomycin stock

For a 1 mM Oligomycin stock, dissolve 5 mg Oligomycin A in 6.32 mL DMSO. Prepare aliquots and store at −20°C. Stock can be used for up to 3 months.

### βES stock

Dissolve βES-HCl powder to 200 mM in equimolar NaOH to neutralize pH. Store at −20°C in aliquots for up to 6 months.

### Cu(II)SO_4_ stock

For a 50 mM Cu(II)SO_4_ stock, dissolve 79.81 mg Cu(II)SO_4_ in 10 mL ddH_2_O. Can be stored at 18°C–20°C for several months.

### THPTA stock

For a 200 mM THPTA stock, dissolve 100 mg THPTA in 1.151 mL ddH_2_O. Store at −80°C in aliquots. Stock can be used for up to 6 months.

### AZDye Azide Plus stock

Dissolve lyophilized AZDye Azide Plus in adequate amount of DMSO for a 1 mM solution. Sonication might be necessary for complete dissolution. Store at −20°C in aliquots. Stock can be used for ≥2 years.

### Fixation buffer

Prepare a 1:8 dilution of 16% methanol-free formaldehyde (w/v) in PBS to obtain a 2% formaldehyde solution. Prepare fresh.

### Permeabilization buffer

Dissolve 0.5 g saponin in 50 mL PBS (1% saponin), and 0.5 g BSA in ∼30 mL PBS. Add 5 mL 1% saponin solution to the BSA/PBS solution, and add PBS until 50 mL. Pass through 0.22 μm filter. Can be stored at 4°C for several weeks.

### Click buffer

Dissolve 13.99 g Tris-HCl and 1.35 g Tris-base (pH 7.4, final concentration 100 mM) in 1 L ddH_2_O. Can be stored at 18°C–20°C for several months.

**Table:** FACS buffer

Reagent	Final concentration	Amount
BSA	1%	5 g
0.5 M EDTA	2 mM	2 mL
PBS	N/A	500 mL
**Total**	**N/A**	**502 mL**

Buffer can be stored at 4°C for up to 3 months.

### Flow cytometer configurations

Flow cytometer configurations are as follows.

**Table:** Beckman Coulter CytoFLEX configuration

Channel	Laser	Bandpass filter
FL1	Blue – 488 nm	525/40
FL2	Blue – 488 nm	585/42
FL3	Blue – 488 nm	610/20
FL4	Blue – 488 nm	690/50
FL5	Blue – 488 nm	780/60
FL6	Red – 638 nm	660/10
FL7	Red – 638 nm	712/25
FL8	Red – 638 nm	780/60
FL9	Violet – 405 nm	450/45
FL10	Violet – 405 nm	525/40
FL11	Violet – 405 nm	610/20
FL12	Violet – 405 nm	660/10
FL13	Violet – 405 nm	780/60

**Table:** Beckman Coulter CytoFLEX LX configuration

Channel	Laser	Bandpass filter
FL1	Blue – 488 nm	525/40
FL2	Blue – 488 nm	610/20
FL3	Blue – 488 nm	690/50
FL4	Y/G - 561 nm	585/42
FL5	Y/G - 561 nm	610/20
FL6	Y/G - 561 nm	675/30
FL7	Y/G - 561 nm	710/50
FL8	Y/G - 561 nm	763/43
FL9	Red – 638 nm	660/10
FL10	Red – 638 nm	712/25
FL11	Red – 638 nm	763/43
FL12	Violet – 405 nm	450/45
FL13	Violet – 405 nm	525/40
FL14	Violet – 405 nm	610/20
FL15	Violet – 405 nm	660/10
FL16	Violet – 405 nm	763/43
FL17	UV – 355 nm	405/30
FL18	UV – 355 nm	675/30
FL19	UV – 355 nm	740/35
FL20	IR – 808 nm	840/20
FL21	IR – 808 nm	885/40

## Step-by-step method details

### CENCAT: Inhibitor and βES treatment

**Timing: 1 h**

The following steps describe the inhibition of metabolic routes for ATP synthesis and subsequent incubation with βES for measuring protein synthesis.

1. During activation/resting of cells, prepare the four inhibitor treatments (10×) in complete medium for the required number of wells.

**Table:** 10× mixes for metabolic inhibition

Condition	Compound 1	Compound 1 stock	Compound 1 dilution for 10×	Compound 2	Compound 2 stock	Compound 2 dilution for 10×
Vehicle control	ddH_2_O	N/A	1:2	DMSO	N/A	1:100
2-DG	2-DG	2 M	1:2	DMSO	N/A	1:100
Oligomycin	ddH_2_O	N/A	1:2	Oligomycin	1 mM	1:100
2-DG + Oligomycin	2-DG	2 M	1:2	Oligomycin	1 mM	1:100

***Note:*** Prepare some extra volume for each inhibitor condition to account for pipetting error. Always prepare fresh in complete RPMI1640.

2. Pellet the cells by centrifugation at 500 RCF for 3 min, discard the supernatant by flicking the plate.
3. Resuspend in 90 μL complete RPMI1640 medium.
4. Treat cells with 10 μL/well of the four inhibitor mixes. Mix by gently shaking plate.
  a. End concentrations:
    i. 2-DG: 100 mM.
    ii. Oligomycin: 1 μM.
    iii. DMSO: 0.1%.
    iv. ddH_2_O: 5%.

***Note:*** Preferably use a small-volume (10 μL) multichannel pipette to add the inhibitors in quick succession.

5. Incubate cells for 15 min at 37°C/5%CO_2_.
6. During incubation, prepare 11× βES working solution (5.5 mM) in medium.
7. Add βES (final concentration 500 μM) in 10 μL total volume/well and gently mix by pipetting.
8. Incubate cells for 30 min at 37°C/5%CO_2._

***Note:*** If the samples are prone to sedimentation, consider performing all incubation steps under continuous agitation at 100 RPM using an orbital shaker.

### CENCAT: Fixation and permeabilization

**Timing: 1.5 h**

Following inhibition of metabolic routes for ATP synthesis and incubation with βES, cells are fixed and permeabilized to allow for the copper-catalyzed azide-alkyne cycloaddition (CuAAC) reaction, which is detailed in the following steps.

***Optional:*** Cells can be transferred to V-bottom plates to improve pelleting.

9. Pellet the cells by centrifugation at 500 RCF for 3 min, discard the supernatant by flicking the plate.
10. Wash the cells with 200 μl PBS/well, pellet the cells (step 9).
11. Resuspend the cells in 50 μL/well fixable viability stain supplemented with Fc receptor block and incubate 10 min at 18°C–20°C in dark.

**Table:** Viability staining mix for human PBMCs

Fluorophore	Marker/reagent	Clone	Dilution
Viakrome 808	Viability	N/A	1:100
N/A	Human TruStain FcX	N/A	1:100
N/A	Truestain Monocyte blocker	N/A	1:20

**Table:** Viability staining mix for mouse peritoneal lavage, eWAT, spleen, lungs, and kidneys

Fluorophore	Marker/reagent	Clone	Dilution
FITC	F4/80	BM8	1:200
Zombie-NIR	Viability	N/A	1:2000
N/A	Mouse TruStain FcX	93	1:100
N/A	Truestain Monocyte blocker	N/A	1:20

**Table:** Viability staining mix for mouse liver

Fluorophore	Marker/reagent	Clone	Dilution
FITC	CLEC2	17D9	1:200
Zombie-NIR	Viability	N/A	1:2000
N/A	Mouse TruStain FcX	93	1:100
N/A	Truestain Monocyte blocker	N/A	1:20

Note/optional: Always prepare fresh in PBS. Antibodies that need to be stained prior to fixation (e.g. anti-mouse CLEC2 and F4/80) and are conjugated to fluorophores that are CuAAC-resistant (i.e. not PE or PerCP and related tandem dyes) can be included in the viability stain. Include TrueStain monocyte blocker to reduce nonspecific binding of PE and PerCP-based tandem dyes to myeloid cells.

12. Add 150 μL PBS/well, pellet the cells.
13. Fix the cells in 200 μL Fixation buffer/well for 15 min at 18°C–20°C, pellet the cells.
14. Wash the cells with 200 μl PBS/well, pellet the cells.

**Pause point:** Plates can be stored in 200 μL PBS at 4°C in the dark, after which the staining procedure can be resumed at a later time point. The maximum storage duration at 4°C depends on several factors, including the sample type, and should be determined by the user.

15. Permeabilize cells for 15 min in 200 μL Permeabilization buffer, pellet the cells.
  ***Optional:*** After permeabilization, samples can be barcoded using (a combination of) fluorescent succinimidyl esters (e.g. CF820-SE) and pooled to save reagents/antibodies in the subsequent steps.
  a. Wash the cells with 200 μL PBS/well, pellet the cells.
  b. Resuspend cells in 50 μL barcode mix diluted in PBS, incubate 5 min at 18°C–20°C in the dark.
  c. Add 150 μL PBS/well, pellet the cells.
  d. Wash the cells with 200 μL PBS/well, pellet the cells.
  e. Pool samples in 100 μL Click buffer, pellet the cells.
16. Wash the cells with 200 μL Click buffer/well, pellet the cells.

### CENCAT: Click reaction and antibody staining

**Timing: 1.5 h**

The following steps describe the CuAAC (‘click’) reaction, antibody staining of surface markers to identify immune cell subsets, and sample acquisition on a flow cytometer. The CuAAC reaction involves the coupling of the alkyne group in βES to a fluorescent azide probe (AZDye 405) in the presence of Cu(I), which is generated by reduction with sodium ascorbate, and the accelerating ligand THPTA.

17. Prepare a 100 mM sodium ascorbate stock by dissolving 19.8 mg sodium ascorbate in 1 mL Click Buffer.

**CRITICAL:** Always prepare fresh before each use to prevent oxidation.

18. Prepare Click Reaction Mix and resuspend the cells in 100 μL Click Reaction Mix/well. Incubate 30 min at 18°C–20°C in dark. After incubation, pellet the cells.

**Table:** Click Reaction Mix

Reagent	Final concentration	Dilution
200 mM THPTA	2 mM	1:100
50 mM Cu(II)SO_4_	500 μM	1:100
100 mM sodium ascorbate	10 mM	1:10
1 mM AZDye 405 Azide Plus	1 μM	1:1000

***Note:*** Always prepare the Click Reaction Mix fresh in Click Buffer, adding the components in the order listed in the table. Add sodium ascorbate and Azide Plus just before applying the Click Reaction Mix to your sample.

19. Wash the cells with 200 μL FACS buffer, pellet the cells.
20. Resuspend the cells in 50 μL antibody staining mix, incubate 15 min at 18°C–20°C in the dark.

**Table:** Post-click antibody staining mix: human PBMCs

Fluorophore	Marker/reagent	Clone	Dilution
BUV395	CD14	MϕP-9	1:200
BUV661	HLA-DR	G46-6	1:200
BUV737	CD56	NCAM16.2	1:800
Spark Violet 500	CD4	SK3	1:400
BV650	CD8	SK1	1:200
AlexaFluor 488	CD62L	DREG-56	1:200
NovaFluor Blue 610-70S	CD3	UCHT1	1:200
PerCP	CD45RA	HI100	1:400
PE	CD123	6H6	1:1600
PE-Cy7	CD11c	S-HCL-3	1:800
APC	TCRγ/δ	11F2	1:400
APC-R700	CD19	HIB19	1:400
APC-Cy7	CD16	3G8	1:400
N/A	Brilliant Stain Buffer Plus	N/A	1:10
N/A	CellBlox Blocking Buffer	N/A	1:20

**Table:** Post-click antibody staining mix: mouse tissue immune cells

Fluorophore	Marker/reagent	Clone	Dilution
FITC	CD4	GK1.5	1:200
PE	TIM-4	RMT4-54	1:200 (1:4000 for peritoneal wash)
PE-Dazzle 594	Siglec-F	S17007L	1:100
PerCP-Cy5.5	CD45	30-F11	1:400
PE-Cy7	CD8a	53–6.7	1:400
APC	CD64	X54-5/7.1	1:400
AlexaFluor 700	Ly6C	HK1.4	1:400
BV510	B220	RA3-6B2	1:100
BV605	CD11c	N418	1:100
BV650	CD11b	M1/70	1:800
BV785	MHCII	M5/114.15.2	1:400
N/A	Brilliant Stain Buffer Plus	N/A	1:10

***Note:*** Always prepare fresh in FACS buffer. Centrifuge antibody mix at 10,000 RCF for 1 min to pellet antibody aggregates.

21. Add 150 μL FACS buffer, pellet the cells.
22. Resuspend cells at ≤20 × 10^6^ cells/mL.
23. Acquire samples on a Beckman Coulter CytoFLEX or CytoFLEX LX, or alternative. Ensure instrument quality control (e.g. using CytoFLEX Ready to Use Daily QC Fluorospheres, Beckman Coulter, cat#C65719), consider instrument standardization and ensure proper panel compensation. Refer to handbooks on high-parameter flow cytometry for details elsewhere.^7^

## Expected outcomes

This protocol describes the metabolic profiling of human peripheral blood and mouse tissue immune cells using CENCAT. Through the PBMC isolation protocol described here, utilizing LeucoSep tubes, we generally isolate ∼10–20 × 10^6^ PBMCs per 10 mL blood. The mouse tissue isolation protocols generally result in the following yields, from 12–16-week old, male C57BL/6J mice.

**Table:** Cell yield from murine tissues

Tissue	Yield (x10^6^)
Mouse peritoneal lavage	0.25–1.00
Mouse eWAT	0.25–1.00
Mouse liver	5–10
Mouse spleen	50–100
Mouse kidney	0.25–1.00
Mouse lung	1–5

For gating strategies of PBMCs and murine tissue immune cells, please refer to Vrieling et al.^1^

**Figure 2:**
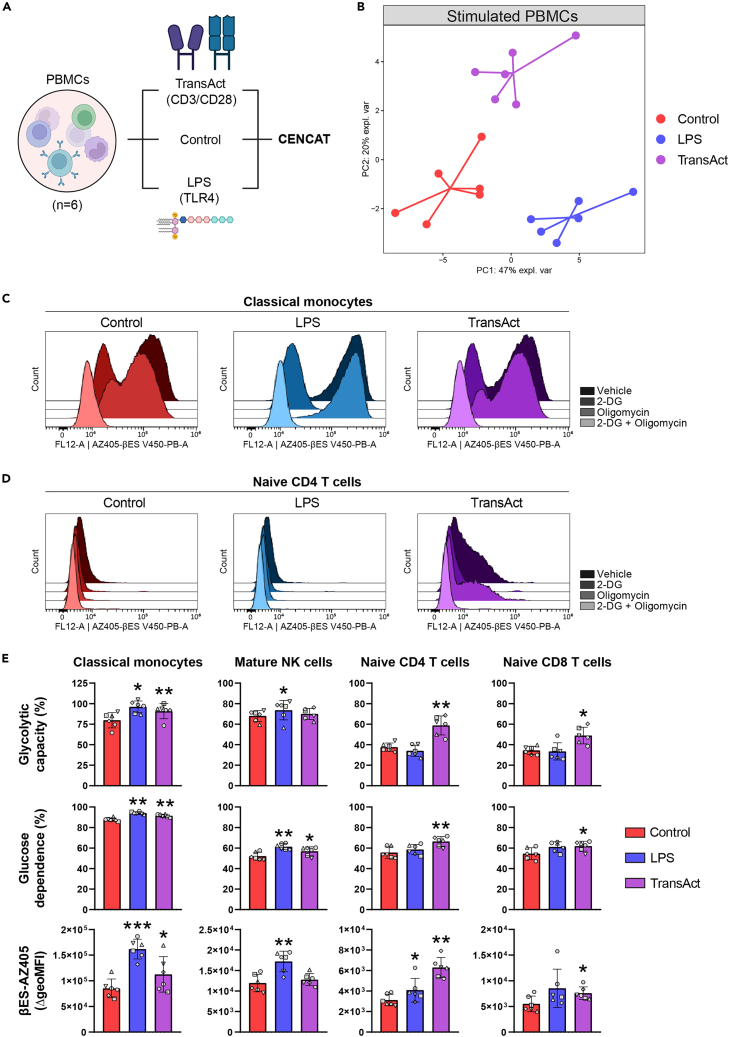
CENCAT analysis of human PBMCs stimulated with LPS, TransAct, or medium control (A) PBMCs were isolated from healthy blood donors (*n* = 6) and subsequently stimulated with LPS (10 ng/mL), TransAct (1:100) or medium control for 2 h, after which cells were subjected to CENCAT analysis. Created with BioRender.com. (B) Multilevel principal component analysis (PCA) score plot (multilevel factor = donor) based on metabolic dependencies of shared immune cell populations. Control samples are depicted in red, LPS in blue, and TransAct in purple. (C and D) Representative histograms of protein synthesis (βES incorporation) for Vehicle, 2-DG, oligomycin, and 2-DG + oligomycin conditions as measured after control (red), LPS (blue) or TransAct (purple) stimulation in (C) classical monocytes and (D) naive CD4 T cells. (E) Mean ± SD glycolytic capacity (%), glucose dependence (%) and βES incorporation (βES-AZ405 ΔgeoMFI) of classical monocytes, mature NK cells, naive CD4 T cells, and naive CD8 T cells treated with medium control (red), LPS (blue) or Transact (purple). Significance was tested by repeated measures one-way ANOVA with Geisser-greenhouse correction and Dunnett’s multiple comparison test. Individual donors are displayed with different symbols. ∗*p* < 0.05, ∗∗*p* < 0.01, and ∗∗∗*p* < 0.001.

We applied CENCAT to analyze the metabolism of human PBMCS after stimulation with LPS or TransAct (Figure 2A). Stimulation resulted in separate clustering of samples based on immune cell metabolic dependencies as visualized by PCA (Figure 2B), indicative of differential metabolic activation by LPS and TransAct compared to the control condition. This differential response is exemplified by classical monocytes (Figure 2C), which showed elevated bES incorporation and reduced inhibition by oligomycin after LPS stimulation compared to medium control, indicative of increased glycolytic capacity and metabolic activity. A similar response was observed in naive CD4 T cells after stimulation with TransAct (Figure 2D). Quantification of metabolic parameters (Figure 2E) confirmed that LPS potently enhanced glycolytic capacity, glucose dependency, and protein synthesis in innate immune cells (e.g., classical monocytes and mature NK cells), whereas TransAct elicited similar metabolic activation in T lymphocytes, including naive CD4 and CD8 T cells. These findings are consistent with the known roles of LPS in activating innate immunity and TransAct in stimulating T cell responses.

**Figure 3:**
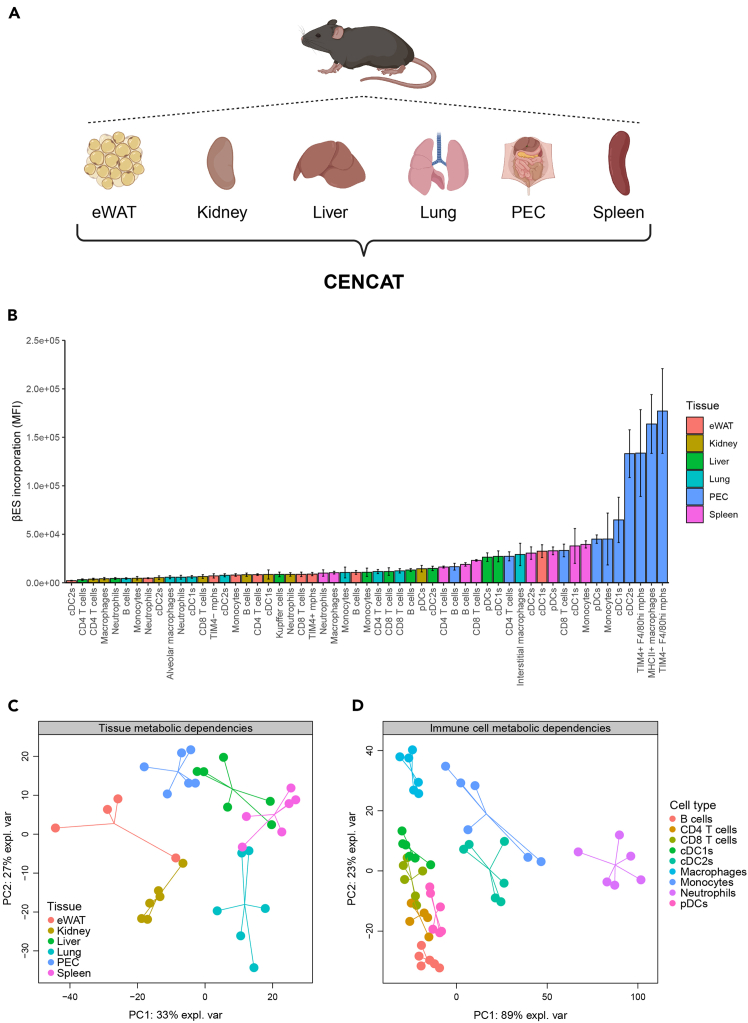
CENCAT analysis of murine tissue-resident immune cell populations (A) The following tissues were isolated from male C57BL/6J mice and subjected to CENCAT analysis: eWAT (red, *n* = 4), kidney (yellow, *n* = 6), liver (green, *n* = 6), lung (cyan, *n* = 6), PECs (blue, *n* = 6), and spleen (pink, *n* = 6). Created with BioRender.com. (B) βES incorporation (MFI) of immune cell populations from all six tissues. Data are displayed as means ± SD. (C and D) Multilevel principal component analysis (PCA) score plots (multilevel factor = mice) based on metabolic dependencies of immune cell populations in all six tissues. Plots were generated based on (C) immune cell metabolic dependencies within tissues and (D) cell type-specific metabolic dependencies across tissues. Figure is reprinted and adapted with permission from Vrieling et al.^1^

Additionally, we employed CENCAT to analyze tissue-resident immune cells in various murine tissues (Figure 3A). βES incorporation was dependent on tissue origin and cell type (Figure 3B), with the highest levels detected in myeloid cells from Peritoneal Exudate Cells (PEC) and spleen. Principal Component Analysis (PCA) based on metabolic profiles of shared immune cell populations between tissues revealed distinct clustering of samples from different tissues (Figure 3C). Similarly, analysis of metabolic profiles of individual immune cell types across tissues also resulted in clear group separation in PCA (Figure 3D). To conclude, CENCAT is a powerful tool to perform metabolic profiling of tissue-resident immune cell populations in mice.

## Quantification and statistical analysis

Calculation of CENCAT parameters is identical to those calculated during SCENITH.^14^ The relative cellular reliance on glucose metabolism and mitochondrial respiration is assessed by comparing the geometric mean fluorescence intensities (geoMFIs) of ncAA labeling under different inhibitor conditions. Glucose dependence (%) is determined by dividing the ΔgeoMFI between DMSO and 2-DG by the ΔgeoMFI between DMSO and the combined 2-DG + oligomycin treatment. Subtracting this value from 100% yields the fatty acid/amino acid oxidation (FAO/AAO) capacity (%). Similarly, mitochondrial dependence (%) is calculated by dividing the ΔgeoMFI between DMSO and oligomycin by the ΔgeoMFI between DMSO and 2-DG + oligomycin. Subtracting this from 100% gives the glycolytic capacity (%).

$$Mitochondrial\;dependence\;\left(\%\right)=\frac{Vehicle-Oligomycin}{Vehicle-2DG+Oligomycin}\times 100$$

$$Glucose\;dependence\;\left(\%\right)=\frac{Vehicle-2DG}{Vehicle-2DG+Oligomycin}\times 100$$

$$Glycolytic\;capacity\;\left(\%\right)=100-\left(\frac{Vehicle-Oligomycin}{Vehicle-2DG+Oligomycin}\times 100\right)$$

$$FAO/AAO\;capacity\;\left(\%\right)=100-\left(\frac{Vehicle-2DG}{Vehicle-2DG+Oligomycin}\times 100\right)$$

For PCA, data was mean-centered and scaled to standard deviation units. Analysis was performed using the pca() function of the mixOmics package in R.

## Limitations

### Low resolution for metabolic profiling in quiescent cells

As with similar tools such as SCENITH,^14^ CENCAT relies on protein synthesis to assess metabolic dependencies. As such, quiescent cells that display low protein synthesis have a smaller window for detecting protein synthesis inhibition, reducing the resolution of functional metabolic profiling.

### Non-specific probe accumulation in eosinophils

Eosinophils are a unique challenge to metabolically profile through CENCAT in its current form, as they display high basal signal intensity for the azide-containing fluorescent probe after the CuAAC reaction, even in the absence of the alkyne-containing βES. While speculative, eosinophils are densely packed with cationic granules, which may result in non-specific probe accumulation. However, CENCAT remains applicable across most immune cell subsets, as this issue is only observed in eosinophils.

### Protein secretion during immune cell activation

**Figure 4:**
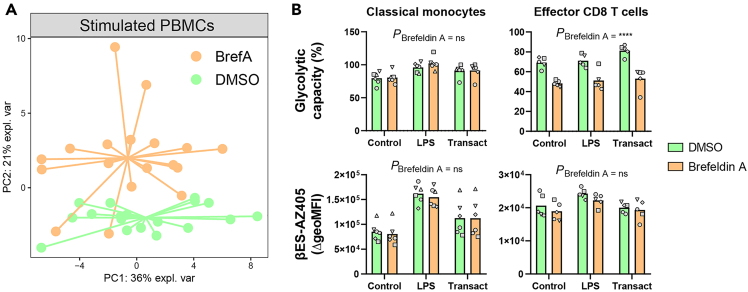
Brefeldin A skews metabolic dependencies in stimulated human PBMCs without affecting protein synthesis PBMCs were isolated from healthy blood donors (*n* = 6) and subsequently stimulated with LPS (10 ng/mL), TransAct (1:100) or medium control for 2 h, after which cells were subjected to CENCAT analysis in the presence of Brefeldin A (5 μg/mL) or DMSO control. (A) Multilevel principal component analysis (PCA) score plot (multilevel factor = donor) based on metabolic dependencies of shared immune cell populations. DMSO-treated samples are depicted in green, Brefeldin A-treated samples in orange. (B) Mean glycolytic capacity (%) and βES incorporation (βES-AZ405 ΔgeoMFI) of classical monocytes, and effector CD8 T cells stimulated with medium control, LPS or Transact, and treated with DMSO (green) or Brefeldin A (orange) during CENCAT. Significance was tested by repeated measures two-way ANOVA with Geisser-greenhouse correction. Individual donors are displayed with different symbols. ∗∗∗∗*p* < 0.0001.

Immune cells activation results in increased secretion of cytokines and other inflammatory mediators, which may potentially confound protein synthesis rates determined via CENCAT. To address this, we tested the use of the protein transport inhibitor Brefeldin A during βES incorporation in human PBMCs stimulated with LPS, TransAct or medium control during 2 h (Figure 4). Strikingly, this treatment did not significantly impact protein synthesis rates compared with untreated control samples, suggesting that these secreted factors have a minor contribution to total protein synthesis. Instead, Brefeldin A treatment resulted in off-target effects on mitochondrial metabolism, particularly in T lymphocytes, thereby skewing metabolic dependencies. We therefore do not recommend including Brefeldin A during βES incorporation in human PBMCs.

### Single-subset resolution

To acquire metabolic profiles through CENCAT, samples are split over four conditions of metabolic inhibition. Since CENCAT is a flow cytometry-based tool, it profiles cells at a single-subset rather than a true single-cell resolution. Hence, temporal assessment of single-cell metabolic profiles is not possible in CENCAT’s current form. However, since CENCAT and SCENITH^14^ allow for measuring metabolic profiles of heterogeneous samples while requiring relatively low cell numbers, they resolve a major limitation of Seahorse extracellular flux analysis.

### Batch effects

We have observed that the purity of leukocytes from tissue samples affects βES incorporation rates. Since the efficiency of cell isolation may vary per experimental day, we recommend including samples from all experimental groups in parallel whenever possible, facilitating batch correction during data analysis.

### Fluorescent quenching due to CuAAC

It has been reported that copper sulfate, in combination with sodium ascorbate, key components of the CuAAC reaction mix, can quench the fluorescence of several fluorochromes, including PE, PE-Cy7, and PerCP. Therefore, we recommend applying any flow cytometry-based immunostaining after the CuAAC reaction when performing CENCAT. If this is not feasible, the addition of fresh THPTA (1 mM) and aminoguanidine (160 mM) to the CuAAC reaction mix has been shown to restore fluorochrome signal intensity.^15^

### β-ethynylserine transporter dependence

The uptake dynamics of βES, including the identity of the involved cellular transporters, have yet to be elucidated. Its incorporation may depend on the expression of specific transport proteins, which can vary between cell types and may be influenced by experimental treatments. As a result, βES incorporation levels should be interpreted with caution when comparing different cell types and/or treatment conditions.

## Troubleshooting

For this section, we limit ourselves to troubleshooting CENCAT, as tissue processing, cell isolation and PBMC activation have been extensively troubleshooted in other STAR protocols (e.g., Liu et al.^3^ and Prosser et al.^6^).

### Problem 1

Low βES signal in basal condition.

### Potential solutions

Low βES signal intensity can be caused by multiple factors. Assuming appropriate equipment set-up and samples were properly incubated with the correct concentration of βES and the appropriate azide-containing AZdye, samples may have been exposed to direct light, causing photobleaching of the fluorochrome. As a solution, ensure incubation steps from step 11 onwards are performed in the dark and prevent direct exposure of samples to light. In addition, suboptimal sample quality, such as low viability or sample impurity, may also reduce βES incorporation. Revisit the isolation protocol and/or consider leukocyte enrichment (e.g. via CD45 MACS) before performing CENCAT. Finally, quiescent cells exhibit low protein synthesis. In such cases, consider activating cells or performing signal amplification using azide-biotin CuAAC followed by incubation with fluorochrome-conjugated streptavidin.

### Problem 2

High βES background signal.

### Potential solution

High βES background signal may result from non-specific accumulation of the fluorescent, as observed for eosinophils (see ‘limitations’). While we currently have no solution for this, it appears to be eosinophil-specific. Other causes may be inadequate equipment settings (e.g., too high channel gains) or improper probe titration. In these cases, consider performing a channel gain titration on the flow cytometer and/or probe titration.

### Problem 3

Negative or >100% values for metabolic dependencies.

### Potential solution

Negative values for metabolic dependencies occur when the βES signal in the 2-DG or oligomycin conditions exceed that of the vehicle control condition. Conversely, values greater than 100% occur when the βES signal in the 2-DG or oligomycin conditions is lower than in the combined 2-DG+oligomycin condition. Conceptually,^14^ negative values reflect no dependence on the inhibited metabolic route for ATP synthesis, whereas >100% dependence indicates complete dependence on these pathways. The non-physiological values are more likely to occur when protein synthesis resolution is low, for instance in quiescent cells or impure samples. Refer to problem 1 in such cases.

### Problem 4

Multiple peaks in protein synthesis histogram.

### Potential solution

Multiple peaks in the βES incorporation histogram indicate heterogeneity within the analyzed subset, displaying different metabolic activity and/or metabolic profiles. As a solution, consider including additional, distinctive cell surface markers into the antibody panel to distinguish relevant subpopulations.

### Problem 5

Poor resolution of cell surface markers and/or FSC/SSC.

### Potential solution

Poor resolution of cell surface markers can result from several factors, including photobleaching, inadequate equipment settings and improper antibody titers (see problem 1 and 2). In addition, formaldehyde fixation may impair epitope recognition by certain antibody clones. Consider testing whether formaldehyde impacts epitope binding for the antibody in question. If this is the case, either stain before fixation or use a different antibody clone that remains effective in epitope recognition after formaldehyde fixation. Lastly, cell shrinkage is commonly observed after fixation, permeabilization, and CuAAC staining, which may reduce resolution of the forward scatter (FSC) and side scatter (SSC) parameters.

Alternatively, if poor resolution occurs for marker stained prior to the fluorescent click reaction, the CuAAC reaction may have quenched the fluorochrome, as has been reported for PE and PerCP-based fluorochromes. In such cases, consider staining for the affected marker after the CuAAC reaction, or including aminoguanidine in addition to THPTA in the click reaction mix, as described by others.^15^

## Resource availability

### Lead contact

Further information and requests for resources and reagents should be directed to and will be fulfilled by the lead contact, Frank Vrieling (frank.vrieling@wur.nl).

### Technical contact

Technical questions on executing this protocol should be directed to and will be answered by the technical contact, Frank Vrieling (frank.vrieling@wur.nl).

### Materials availability

This study did not generate new unique materials. However, βES-HCl is available from the lab of Dr. Kimberley Bonger (Leiden Institute of Chemistry, Leiden University, the Netherlands) upon request.

### Data and code availability

All data reported are available from the lead contact upon request. This protocol did not generate any new code.

## Acknowledgments

F.V. was partly funded by a research fellowship from the European Society for Clinical Nutrition and Metabolism (ESPEN). H.J.P.v.d.Z. and R.S. were partly funded via the consortium The Right Timing to Prevent Type 2 Diabetes (TIMED), funded by the Netherlands Organization for Health Research and Development (ZonMw) (459001021), the Dutch Diabetes Research Foundation (Diabetes Fonds) (2019.11.101), the Canadian Institutes of Health Research (CIHR) (TNC-174963), and Health-Holland (LSHM20107). We greatly appreciate the collaboration with Dr. Kimberly Bonger (Leiden Institute of Chemistry, Leiden University, the Netherlands) for providing crucial reagents. The graphical abstract was created with BioRender.com.

## Author contributions

F.V. conceived the study. F.V. and H.J.P.v.d.Z. performed experiments, analyzed data, and wrote the manuscript. M.D. performed experiments. R.S. supervised the study and reviewed the manuscript.

## Declaration of interests

The authors declare no competing interests.

## References

[1] Vrieling F., van der Zande H.J.P., Naus B., Smeehuijzen L., van Heck J.I.P., Ignacio B.J., Bonger K.M., Van den Bossche J., Kersten S., Stienstra R. (2024). CENCAT enables immunometabolic profiling by measuring protein synthesis via bioorthogonal noncanonical amino acid tagging. Cell Rep. Methods.

[2] van der Zande H.J., Brombacher E.C., Lambooij J.M., Pelgrom L.R., Zawistowska-Deniziak A., Patente T.A., Heieis G.A., Otto F., Ozir-Fazalalikhan A., Yazdanbakhsh M. (2023). Dendritic cell-intrinsic LKB1-AMPK/SIK signaling controls metabolic homeostasis by limiting the hepatic Th17 response during obesity. JCI Insight.

[3] Liu Z., Gu Y., Shin A., Zhang S., Ginhoux F. (2020). Analysis of Myeloid Cells in Mouse Tissues with Flow Cytometry. STAR Protoc..

[4] Hearnden R., Sandhar B., Vyas V., Longhi M.P. (2021). Isolation of stromal vascular fraction cell suspensions from mouse and human adipose tissues for downstream applications. STAR Protoc..

[5] Petry P., Aktories P., Oschwald A., Kierdorf K. (2024). Protocol for long-term monocultures of murine macrophages derived from distinct adult tissues. STAR Protoc..

[6] Prosser A., Dart S., Larma-Cornwall I., Lucas M. (2021). Flow cytometric characterization of tissue-resident lymphocytes after murine liver and heart transplantation. STAR Protoc..

[7] Mair F., Tyznik A.J. (2019). High-Dimensional Immunophenotyping with Fluorescence-Based Cytometry: A Practical Guidebook. Methods Mol. Biol..

[8] Ray A., Dittel B.N. (2010). Isolation of mouse peritoneal cavity cells. J. Vis. Exp..

[9] Andreata F., Blériot C., Di Lucia P., De Simone G., Fumagalli V., Ficht X., Beccaria C.G., Kuka M., Ginhoux F., Iannacone M. (2021). Isolation of mouse Kupffer cells for phenotypic and functional studies. STAR Protoc..

[10] Wickham H. (2016).

[11] Wilke C.O. (2025). cowplot: Streamlined Plot Theme and Plot Annotations for 'ggplot2'. https://wilkelab.org/cowplot/.

[12] van den Brand T. (2025). ggh4x: Hacks for 'ggplot2'. https://github.com/teunbrand/ggh4x.

[13] Rohart F., Gautier B., Singh A., Lê Cao K.A. (2017). mixOmics: An R package for 'omics feature selection and multiple data integration. PLoS Comput. Biol..

[14] Arguello R.J., Combes A.J., Char R., Gigan J.P., Baaziz A.I., Bousiquot E., Camosseto V., Samad B., Tsui J., Yan P. (2020). SCENITH: A Flow Cytometry-Based Method to Functionally Profile Energy Metabolism with Single-Cell Resolution. Cell Metab..

[15] Pelgrom L.R., Davis G.M., O'Shaughnessy S., Wezenberg E.J.M., Van Kasteren S.I., Finlay D.K., Sinclair L.V. (2023). QUAS-R: An SLC1A5-mediated glutamine uptake assay with single-cell resolution reveals metabolic heterogeneity with immune populations. Cell Rep..

